# Extracellular vesicles recovered from plasma of severe dengue patients induce CD4+ T cell suppression through PD-L1/PD-1 interaction

**DOI:** 10.1128/mbio.01823-23

**Published:** 2023-11-20

**Authors:** Sharda Kumari, Bhaswati Bandyopadhyay, Anamika Singh, Suruchi Aggarwal, Amit Kumar Yadav, Naval Kishore Vikram, Prasenjit Guchhait, Arup Banerjee

**Affiliations:** 1 Laboratory of Virology, Regional Centre for Biotechnology, NCR Biotech Science Cluster, Faridabad, India; 2 Department of Microbiology, Calcutta School of Tropical Medicine, Kolkata, West Bengal, India; 3 Disease Biology Laboratory, Regional Centre for Biotechnology, NCR Biotech Science Cluster, Faridabad, India; 4 Translational Health Science and Technology Institute, NCR Biotech Science Cluster, Faridabad, Haryana, India; 5 Department of Infectious Disease & Medicine, All India Institute of Medical Sciences, New Delhi, India; Johns Hopkins Bloomberg School of Public Health, Baltimore, Maryland, USA

**Keywords:** extracellular vesicles, CD4 proliferation, immunosuppression, dengue, PD-1/PD-L1

## Abstract

Extracellular vesicles (EVs) are small membrane vesicles secreted into biological fluids, which play crucial roles in influencing the cellular function in different pathological processes. However, there is less clarity on how plasma EVs influence proliferation activation and functions of immune cells during dengue virus (DV) infection. In this study, we aimed to characterize circulating EVs from different categories of dengue patients and examined the consequence of naïve CD4+ T cells to EVs isolated from the plasma of mild or severe dengue patients. We observed that severe dengue infection was associated with an increased release of CD41a+ platelet extracellular vesicles compared with that from patients with mild disease or healthy donors. These EVs carried an increased level of pro- and anti-inflammatory cytokines along with immunoregulatory proteins on the surface that caused CD4+ T cell suppression. Treatment of purified naïve CD4+ T cells with EVs derived from severe dengue plasma drove CD4+ proliferation toward specific subtypes and modulated surface receptor CXCR3 and CCR6 expression. Subsequent studies indicated a notable rise in programmed cell death ligand 1 (PD-L1) expression within CD41a+ severe DV (SDV) EVs. Additionally, CD4+ T cells showed an elevated increase in programmed cell death 1 (PD-1) after incubating with SDV-EVs. We also demonstrated that blocking PD-L1 on SDV-EVs or PD-1 on late-activating CD4^+^ T cells partially reversed the SDV-EV-induced suppression of CD4+ T cell proliferation. Overall, our study highlights the immunosuppressive property of EVs derived from plasma of severe dengue patients, which might contribute to immune pathogenesis by shaping the immune response.

**IMPORTANCE:**

Severe dengue manifestations caused by the dengue virus are a global health problem. Studies suggest that severe dengue disease depends on uncontrolled immune cell activation, and excessive inflammation adds to the pathogenesis of severe dengue disease. Therefore, it is important to understand the process that triggers the uncontrolled activation of the immune cells. The change in immune response in mild to severe dengue may be due to direct virus-to-cell interaction or it could be a contact-independent process through the extracellular vesicles (EVs) released from infected cells. The importance of circulating EVs in the context of dengue virus infection and pathogenesis remains unexplored. Therefore, understanding the possible biological function of circulating EVs may help to delineate the role of EVs in the progression of disease. Our present study highlights that EVs from plasma of severe dengue patients can have immunosuppressive properties on CD4+ T cells which may contribute to T cell suppression and may contribute to dengue disease progression.

## INTRODUCTION

Dengue virus (DV) transmitted by *Aedes* spp. mosquitoes is the most important arthropod-borne disease worldwide (1). Dengue affects 50–100 million people annually, and 40% of the world’s population is at risk (2). Dengue starts as a febrile disease with headache, nausea, and vomiting and occasionally evolves into dengue hemorrhagic fever (DHF) with severe bleeding and organ impairment. Severe dengue leads to dengue shock syndrome (DSS) when plasma leakage along We evaluated some of the pro-inflammatory with a rapid fall in platelet count leads to multi-organ failure and the patient dies due to hypovolemic shock. The development of DHF and DSS depends on many factors (3) and differences between viral strains (4). There are many reports available suggesting that severe dengue disease depends on uncontrolled immune cell activation, and excessive inflammation adds to the pathogenesis of severe dengue disease (5–7). Therefore, it is important to understand the process that triggers the uncontrolled activation of the immune cells. The change in immune response in mild to severe dengue may be due to direct cell-to-cell interaction or it could be a contact-independent process through the extracellular vesicles (EVs) released from infected cells.

EVs are small membrane structures (size range up to 30–200 nm) that are produced by most cells and can be detected in several body fluids. EVs are biosynthesized in the cells through an endocytic pathway where the cargo (DNA, RNA, miRNA, proteins) is packed by the endosomal complexes required for transport (ESCRT)-dependent or independent process (8, 9). EV cargo can be functional in recipient cells after transfer (10). Due to this unique property, EVs have recently received considerable attention. EVs have initially been thought to involve cell-to-cell communication. However, studies reported in the past years showed their vital role under disease conditions. It was previously shown that the EV composition changes drastically during infection, especially infections by intracellular pathogens such as viruses (11). EVs can affect recipient cells either by delivering the cargo into the recipient cells or by interacting with receptors present on the surface and triggering intracellular signaling. It is also reported that EVs released from virus-infected cells can either block or trigger antiviral responses and cytokine secretion even in uninfected cells near the infection site, helping to fight the infection and protect other cells from the subsequent virus infection (12, 13). However, this protective response sometimes can backfire, when a massive inflammation facilitated by those EVs causes tissue damage leading to clinical adverse outcomes (14). EVs released from virus-infected cells can carry viral RNA or proteins and have a covering of plasma membrane which helps them to escape from immune surveillance, thus contributing to the immune evasion process (15). EVs can activate immune cells by the presentation of antigens to their surface (16). They are released from the infected cells and can be circulated in body fluid and may alter disease manifestations.

To date, most studies of vesicle-mediated immune suppression were performed with EVs isolated from *in vitro* cultured supernatants of immune cells or cell lines infected with viruses and, less frequently, from plasma of infected patients (17). The importance of EVs in the context of dengue virus infection and pathogenesis is reported in a few studies (12, 18, 19). Therefore, understanding the possible biological function of circulating EVs may help to delineate the role of EVs in the progression of dengue disease.

In our study, we isolated EVs from severe dengue patients’ plasma (SDV-EVs) and compared their cargo and biological functions against the EVs isolated from plasma of mild dengue patients (MDV-EV), other febrile illness (OFI-EVs), and healthy donors’ (HD-EVs). We observed that severe dengue infection was associated with an increased release of CD41a+ platelet EVs compared with that from patients with mild disease or healthy donors. SDV-EVs also carry high levels of pro- and anti-inflammatory cytokines. Further studies confirm that SDV-EVs carry programmed cell death ligand 1 (PD-L1), which mediates T cell suppression and may be linked to disease severity.

## RESULTS

### Severe dengue is associated with increased release of platelet extracellular vesicles in plasma

**Fig 1:**
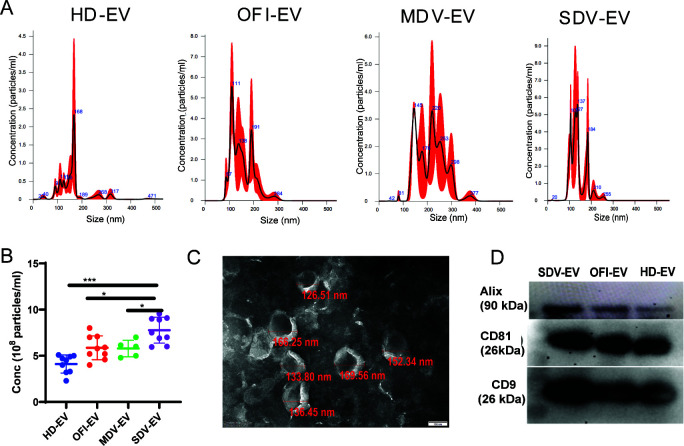
Characterization of EVs isolated from plasma. (**A**) The EVs from plasma were purified by differential ultracentrifugation method. The concentration of purified EVs was determined by nano tracking analysis capturing the Brownian motion of EVs in solution. Particle size distributions were obtained by NanoSight LM20. (**B**) The combined data for different EV donors in each cohort are shown. Each dot represents one set of EVs. Brown-Forsythe and Welch analysis of variance (ANOVA) test was used (*P = 0.033). (**C**) Morphological analysis of plasma EVs was performed by transmission electron micrograph. EVs are in cup-shaped morphology at 80-kV threshold and scale of 100 nm. These EVs appeared in size ranging from 30 nm to 200 nm. (**D**) Molecular protein profiles of plasma EVs from three groups. Western blot of EVs was performed after loading 20 µg protein, and Alix, CD81, and CD9 were checked. HD-EV, EV derived from healthy donors; OFI-EV, EV derived from other febrile illness patients; MDV-EV, EV derived from mild dengue patients; and SDV-EV, EV derived from severe dengue patients.

EVs were prepared from the pooled plasma using differential ultracentrifugation methods (20). We characterized the EVs based on the guidelines issued by the International Society for Extracellular Vesicles (21). To evaluate the size distribution, isolated EVs from different groups were subjected to nanoparticle tracking analysis (NTA) (Fig. 1A). The size of vesicles was in the range of 70–200 nm, with a peak at 147 nm and a mean diameter at 160 nm (mean ± SD; HD-EVs 168 ± 25.7 nm, OFI-EVs 111.5 ± 20.5, MDV-EVs 145 ± 10 nm, and SDV-EVs 137 ± 8.6 nm). The average particle count was highest in SDV patients (7.2 × 10^8^ particles/mL), intermediate in OFI and mild dengue patients (5.5 × 10^8^ particles/mL), and lowest in HD specimens (3.3 × 10^8^ particles/mL). These differences in particle concentration levels are significant for all cohorts, as compared to HD (Fig. 1B).

The morphology of single vesicles was assayed by transmission electron microscopy (TEM) (Fig. 1C). Using TEM, we observed a cup-shaped morphology corresponding to the EV size and maintaining the integrity of single vesicles. We also confirmed the presence of exosome markers (Alix, CD81, and CD9) on the surface of vesicles by Western blot (Fig. 1D). Thus, the nanovesicles isolated from human plasma had a typical spherical form, contained a bilayer membrane, and were enriched with exosomal markers.

**Fig 2:**
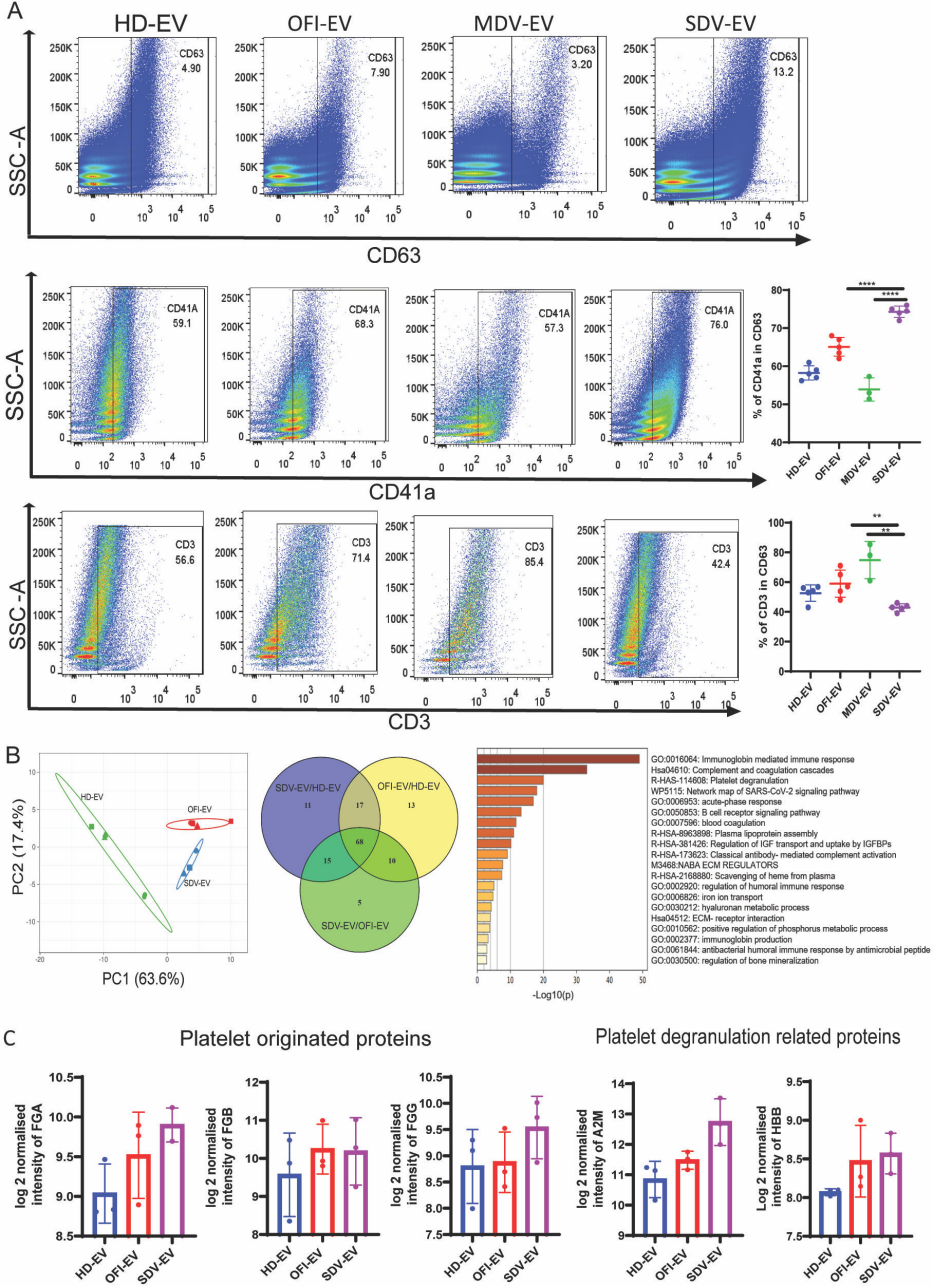
Source of plasma-derived EVs. EVs derived from HD, OFI, mild, and severe dengue patients were checked for the (**A**) presence of CD63 (EVs marker), CD41a (platelet marker), and CD3 (T cell marker) by flow cytometry. EVs were first gated for the CD63+ population (upper panel). Within the CD63+ population, CD41a (middle panel) and CD3 population were further gated (lower panel). Percent CD41a+ and CD3+ populations from multiple sets were plotted (right panels). Each dot represents an individual set of EVs used for the experiment. HD (*n* = 5), OFI (*n* = 5), mild DV (*n* = 3), and severe dengue (*n* = 5). Unpaired Student *t*-test was used to calculate the *P*-value (****P < 0.0001; **P = 0.001). (**B**) Proteomic profiling of EV lysates. (Left panel) Principal component analysis (PCA) based on all proteins detected in EV lysates. PCA plot demonstrates distinct separation between EVs isolated from different groups. Data were obtained from three sets of EVs from each group. (Middle panel) Venn diagram showing common and unique proteins found in different groups as indicated. (Right panel) Bar diagrams represent the pathways associated with common and unique proteins. (**C**) Proteins related to platelet origin and their degranulation pathway are shown. Data are available via ProteomeXchange with identifier PXD045894. HD-EV, EV derived from healthy donors; OFI-EV, EV derived from other febrile illness patients; MDV-EV, EV derived from mild dengue patients; and SDV-EV, EV derived from severe dengue patients.

Since EVs derived from the plasma may come from various cells dysregulated during dengue infection, we further looked into the source of the EVs isolated from different groups of patients. We first gated the CD63+ population which is a specific maker for the extracellular vesicles (Fig. 2A). Within the CD63+ population, we then checked platelet marker CD41a and T cell marker CD3 (Fig. 2, middle and lower panels). We observed that about two-thirds of the CD63+ SDV-EVs are also positive for a platelet marker CD41a, suggesting that platelets are the major contributors of EVs in the plasma of severe dengue patients (Fig. 2A, middle panel). On the contrary, mild, OFI, and HD plasma mostly carry EVs released from T cells (Fig. 2A, lower panel). The platelet origin of plasma EVs in severe dengue patients was further supported by our mass spectrometry (MS) data (PXD045894) (Fig. 2B). The principal component analysis (PCA) plot showed a clear distinction between HD, OFI, and SDV patients (left panel). Venn diagram (middle panel) revealed that 68 proteins were found to be common. Gene ontology and pathway enrichment studies further confirmed that the majority of the proteins in SDV-EVs and OFI-EVs were found to be involved in the activation of an immune response, complement and coagulation cascades, and platelet degranulation process (Fig. 2B, right panel). The quantitative values of some platelet-originated proteins were shown in the graphs (FGA, FGB, and FGG) (22), and the proteins involved in platelet degranulation processes were shown (A2M and HBB) in Fig. 2C. Overall, our data suggested that severe dengue infection is associated with increased release of platelet extracellular vesicles in plasma.

### SDV-EVs carry both pro and anti-inflammatory cytokines

EVs isolated from DV-positive and DV-negative patients may carry different sets of cargo. To examine the variation in cytokine levels within different EV groups, we performed a multiplex assay using the FACS-based 12-plex panel. EVs and their corresponding plasma were used for the assay. We evaluated some of the pro-inflammatory (IFNγ, IL-6, TNF-α, IL-17a, and IL-2) and anti-inflammatory (IL-13, IL-5, IL-4, IL-10, and IL-22) cytokines in the patient’s plasma as well as in EVs derived from them. These cytokines are also reported in dengue viral infection and inflammation (23, 24).

**Fig 3:**
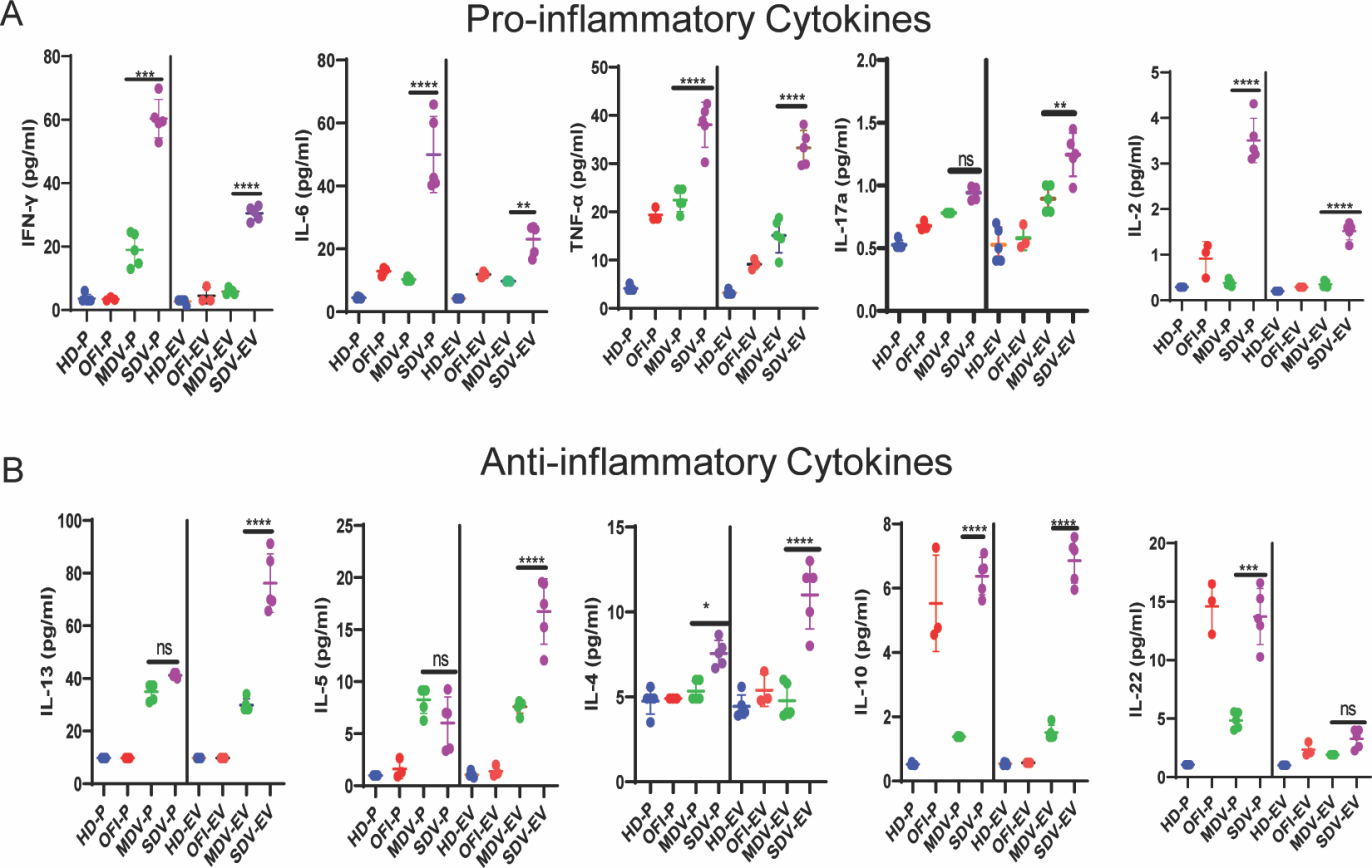
Cytokine array of extracellular vesicles. Comparison of cytokine expression in EVs using cytokine bead array (CBA) through flow cytometry. (**A**) Pro-inflammatory (IFNγ, IL-6, TNF-α, IL17a, and IL-2) (**B**) anti-inflammatory cytokines (IL-13, IL-4, IL-5, IL-10, and IL-22) were detected in the plasma obtained from all four groups, and EVs were derived from them. Each dot represents a pool of five samples. The pattern of cytokines in EVs was in synchronization with the plasma of their respective groups. One-way ANOVA with multiple comparisons was used to obtain the significance (****P < 0.0001; **P = 0.0039). HD (*n* = 5), OFI (*n* = 3), mild DV (*n* = 5), and severe dengue (*n* = 5). HD-EV, EV derived from healthy donors; OFI-EV, EV derived from other febrile illness patients; MDV-EV, EV derived from mild dengue patients; and SDV-EV, EV derived from severe dengue patients. P represents the plasma of respective groups.

Analysis of individual cytokines in EVs revealed that eight cytokines, IFNγ, TNF-α, IL-2, IL-6, IL-17a, IL-13, IL-5, and IL-4, were found in greater levels in SDV-EVs and their corresponding plasma than in OFI-EVs and MDV-EVs (Fig. 3A and B). Although IL-10 level was significantly higher in SDV-EVs than in OFI-EVs, no difference was observed in plasma levels. Between mild and severe dengue groups, IL-10 levels were significantly elevated in severe dengue, both in plasma and EVs. Similarly, IL-22 level increased in OFI and SDV plasma than in HD, but no significant differences were observed in their expression level in EVs of SDV and OFI. Among the pro- and anti-inflammatory cytokines, IL-13 was highly enriched in SDV-EVs followed by TNF-α, IFNγ, IL-6, and IL-5. Plasma and EVs of healthy donors showed a negligible amount of all these pro- and anti-inflammatory cytokines (Fig. 3).

### SDV-EVs drive CD4+ proliferation toward specific subtypes and affect cytokine production

**Fig 4:**
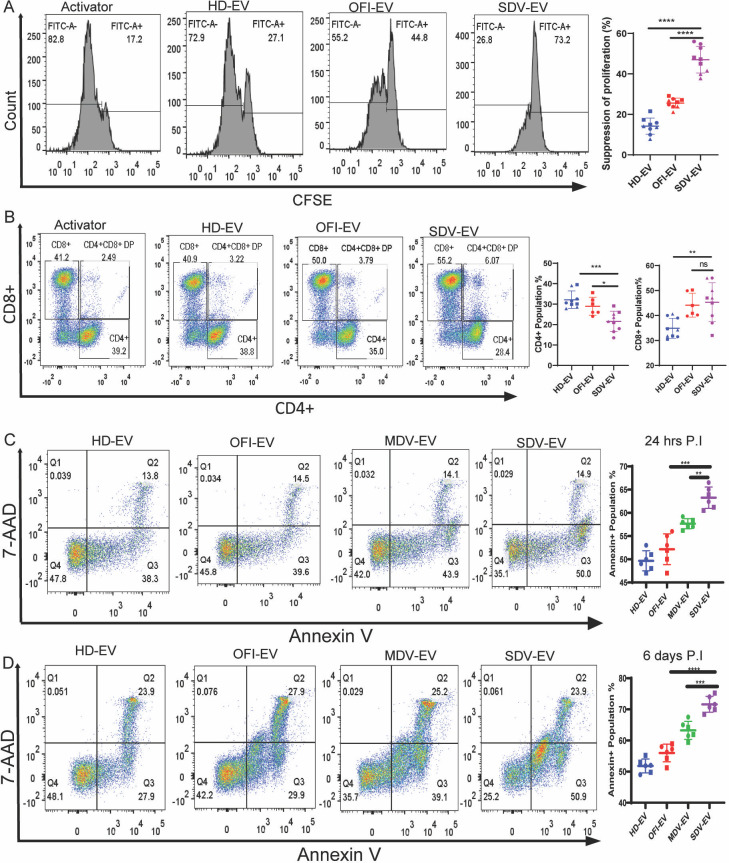
EV-mediated suppression of PBMC proliferation. (**A**) PBMCs were activated with CD3:CD28 antibody and cultured with and without EV for 6 days. Representative histograms of CFSE+ PBMC proliferation in the presence and absence of respective EVs from the plasma of patients and healthy donors were shown. Suppression of proliferation was calculated by subtracting the value of the activator from the respective groups. Combined data of CFSE proliferation on three individual donors with EVs showed significant suppression of proliferation in SDV-EV (right panel). HD-EVs (*n* = 9), OFI-EVs (*n* = 6), and SDV-EVs (*n* = 9). Student unpaired two-tailed test was used to observe the *P*-value (****P < 0.0001). (**B**) Representative contour plot of CD4+ and CD8 distribution in PBMC in CD3+ gated population. PBMC was cultured with and without EVs from three cohorts for 6 days. The percent CD4+ and CD8+ T cell population was plotted (right panel). The combined graph representing data from three individual donors shows the decreased number of CD4+ T cells in the presence of SDV-EVs as compared with OFI-EVs and HD-EVs, but no significant changes in CD8+ T cells. HD-EVs (*n* = 9), OFI-EVs (*n* = 6), SDV-EVs (*n* = 9). (***P = 0.0002; **P = 0.002; *P = 0.0114). (**C**) Representative plot of apoptosis assay 24 hours post incubation and (**D**) 6 days post incubation with EVs from all four different groups on CD4+ T cells cultured with activator. Percentage of Annexin V+ population plotted (right panel). The combined graph represents data from two individual donors showing increased apoptosis in the presence of SDV-EV as compared with other EVs. Each dot represents one set of EVs, and each color-coded shape represents data of individual donors. Student unpaired *t*-test was used for calculating the significance (**P = 0.003; ***P < 0.001; ****P < 0.0001).

EVs derived from the plasma of severe DV patients may have an immunomodulatory role. To test our hypothesis, we co-incubated EVs isolated from the plasma of SDV and OFI patients or HD with normal pre-activated peripheral blood mononuclear cells (PBMCs), and the proliferation of T cells ± EVs was measured in carboxyfluorescin succinimidyl ester (CFSE)-based assays. Representative data shown in Fig. 4A indicate that SDV-EVs induced the strongest suppression of proliferation (~74%). OFI-EVs induced less suppression (~44%), and HD-EVs exerted the least inhibitory (~27%) effect. We also checked the percent population of CD4+ and CD8+ T cells in PBMC after 6 days of incubating with the EVs from all three groups. A representative gated population of CD4+ and CD8+ cells was shown in Fig. 4B. There was a significantly lesser percentage of CD4+ T cells observed in the case of SDV-EVs, but no significant change in the CD8+ T cells was observed in SDV-EVs and OFI-EVs (Fig. 4B, right panel).

The less CD4 population may be due to an increase in apoptosis. Therefore, we checked the apoptosis after purifying the CD4+ T cells and incubated them in the presence of EVs. The percent apoptotic population was measured by FACS at 24 hours and 6 days post incubation (Fig. 4C and D). The increased apoptotic population was observed in MDV and SDV compared to HD and OFI-EVs at both time points. Between MDV and SDV-EVs, SDV-EV-treated CD4+ T cells exhibited a significant increase in apoptosis than MDV-EVs (Fig. 4C and D, right panel), suggesting that a decrease in CD4+ T cell population is indeed due to an increase in apoptosis.

**Fig 5:**
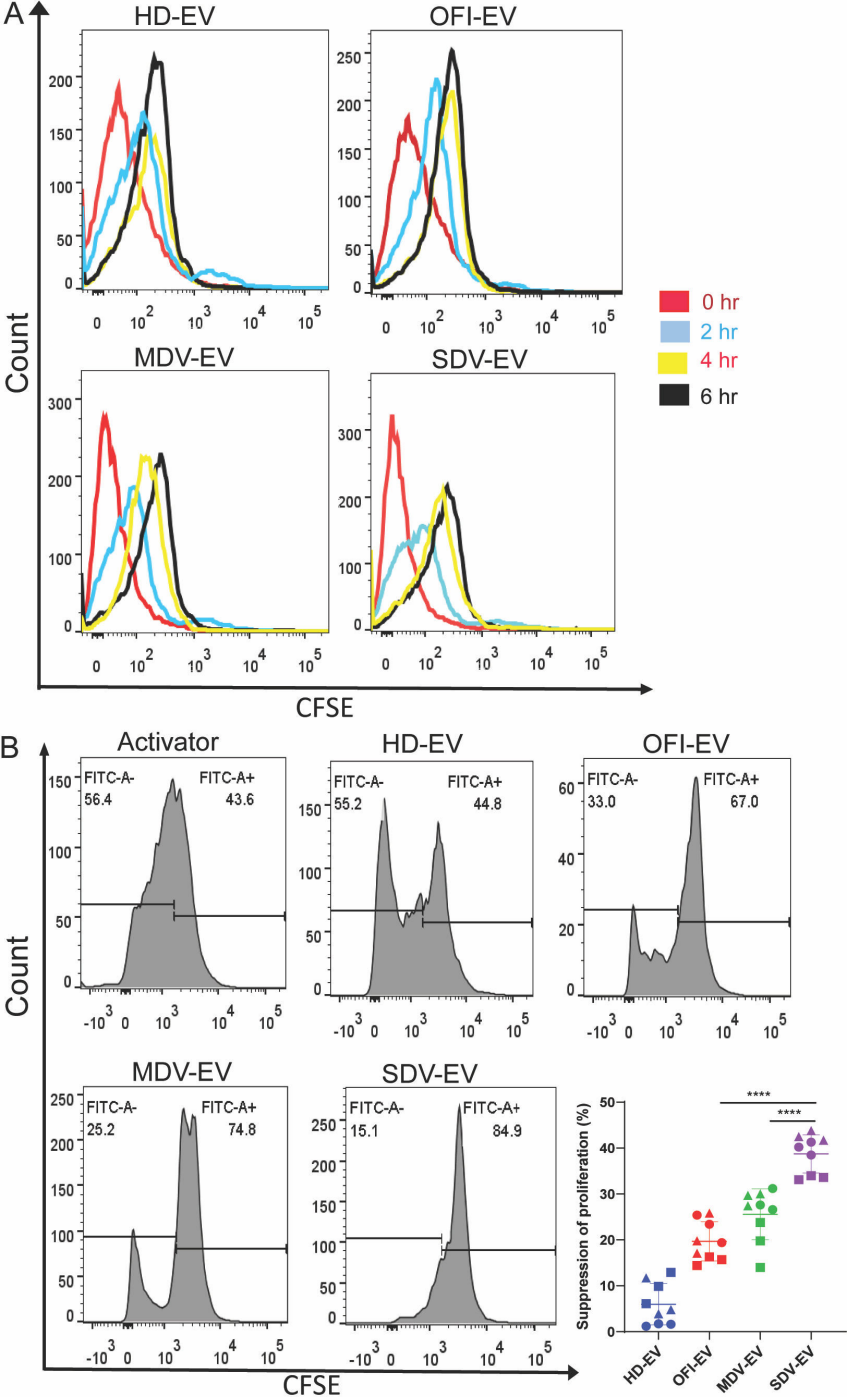
EV-mediated suppression of CD4+ T cell proliferation. (**A**) EVs (20 µg/mL protein equivalent) were labeled with CFSE and incubated with CD4+ T cells. Representative histogram measures the intensity of CFSE detected in CD4+ T cells after the addition of EVs from different cohorts and at four time points (0, 2, 4, 6 hours). The red line demonstrates the fluorescence intensity of cells just after the addition of labeled EVs in CD4+ T cells. (**B**) Activated CD4+ T cells cultured with and without EVs derived from all four cohorts for 6 days. Representative histograms of proliferating CFSE+ CD4+ T cells incubated with EVs were shown. Combined data of CFSE+ CD4+ proliferation on three individual healthy blood donors. Significant suppression of proliferation was observed in SDV-EV as compared to MDV-EV, OFI-EV, and HD-EV (bottom right panel). Suppression of proliferation was calculated by subtracting the value of the activator from the respective groups. Each dot represents data of one donor by one individual set of EVs. HD (*n* = 9), OFI (*n* = 9), mild dengue patients (*n* = 9), and severe dengue (*n* = 9). Each color-coded shape represents data of individual donors. Student unpaired two-tailed test was used to observe the *P*-value. (**** *P* < 0.0001).

We next evaluated how the different EVs interacted with CD4+ T cells. We incubated freshly isolated CD4+ T cells at different time points with the EVs of all four groups labeled with CFSE and analyzed them by flow cytometry (Fig. 5A). The majority of CD4+ T cells became CFSE+ upon treatment with each of the different EVs, with almost similar percentages of CFSE+ cells observed in all conditions (Fig. 5A), suggesting that CD4+ T cells uptake all four groups EVs with almost equal efficiency. There was a gradual shift observed over time, suggesting uptake of EVs by the CD4+ T cells.

Since we observed a decrease in the CD4+ population upon incubation of PBMC with SDV-EVs, we wanted to understand at what levels SDV-EVs act on the CD4+ population. PBMCs also contain other cell population that might affect the results. So, we purified CD4+ T cells, labeled them with CFSE, and activated them with CD3:CD28 incubated with EVs from four groups, and proliferation was measured by the reduction of CFSE dye through flow cytometry. Similar to our PBMC data, SDV-EVs induced strong suppression in purified CD4+ T cells up to 84%, MDV-EVs showed 74%, whereas OFI-EVs showed 67% and HD-EVs showed the least inhibition at 44% (Fig. 5B). A percentage of the suppressive population was plotted after subtracting values from activators, as shown in Fig. 5B, lower right panel. The percentage of CD4+ T cell suppression with SDV-EVs is significantly higher than EVs isolated from mild DV and OFI (Fig. 5B, lower right panel).

Since activation of CD4+ T cells can result in the release of different cytokines, which reflect the polarization state of the T cells, we examined the CD4+ T cell cytokine profile after 6 days of culture with allogeneic EVs. IFNγ is the main cytokine secreted by Th1 effector cells involved in cell-mediated immunity, and IL-13, as well as IL-5 and IL-4, is preferentially produced by differentiated Th2 cells that regulate antibody-mediated humoral immune response (25).

**Fig 6:**
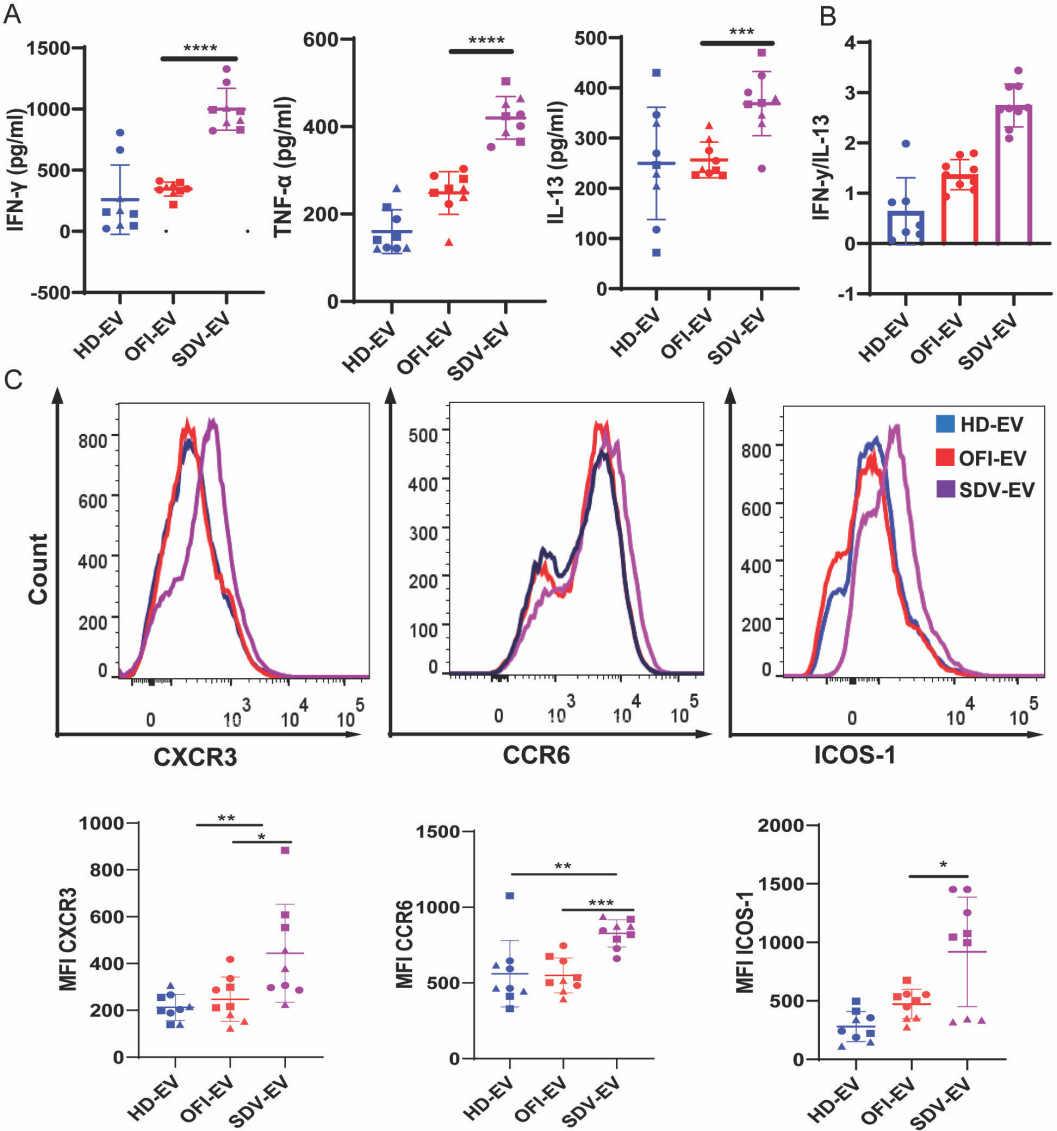
Subset of CD4+ T cells based on cytokines and cell surface markers. (**A**) Cytokines were checked in the supernatant of CD4+ T cells cultured with and without respective EVs. The concentrations of IFNγ, TNF-α, and IL-13 were found to be significantly higher in the presence of SDV-EVs. (**B**) The ratio of IFNγ and IL-13 was plotted for each group. (**C**) Representative data of mean fluorescence intensity (MFI) of CXCR3, CCR6, and ICOS-1 after culturing CD4+ T cells with EVs for 6 days and stained with CXCR3, CCR6, and ICOS-1. The MFI of these markers was found to be increased in the presence of SDV-EVs. Combined data graphs of MFI of CXCR3, CCR6, and ICOS-1 in three individual donors were represented in the lower panel. HD (*n* = 9), OFI (*n* = 6), and SDV-EV (*n* = 9). Student unpaired two-tailed test was used to observe the *P*-value (*P = 0.01; **P = 0.003; ***P < 0.001).

Culture supernatants were collected to assess the production of inflammatory cytokines following incubation with EVs. The production of pro-inflammatory cytokines, IFNγ and TNF-α, by CD4+ T cells (Fig. 6A) was strikingly induced by exposure to SDV-EVs as compared to HD-EVs and OFI-EVs. On the other hand, IL-13 levels increased in SDV-EV-treated groups. However, we did not see significant changes in IL-22, IL-17A, and IL-2 cytokines between SDV-EV- and HD-EV-treated groups (data not shown). We also measured other cytokines in the supernatants, such as IL-9 (Th-9-secreted cytokine) and IL-10 (secreted by Treg), but we did not observe any significant differences compared to their basal levels secreted by non-activated T cells (data not shown).

IFNγ and IL-13 were the two cytokines produced in the greatest amounts in our cultures upon EV treatment (ranges of 500–1,200 ng/mL). We thus used the ratio of secreted IFNγ versus IL-13 as an indication of the relative proportions of Th1 versus Th2 lymphocytes (26). The median IFNγ/IL-13 ratio was below 1 (0.65) for the HD-EV-stimulated CD4+ T cells, whereas it was above 1 (1.5 and 2.5) for the OFI and SDV-EV-stimulated T cells, respectively (Fig. 6B). This suggests that HD-EVs can tip the balance between Th1 and Th2, whereas SDV-EVs tip it toward Th1 lymphocytes.

Since we observed differential expression of cytokines in SDV-EV-treated activated CD4+ T cells, we also looked into the status of T cell subtypes that are most affected in the presence of SDV-EVs.

It is well established that the differential expression profile of CXCR3 and CCR6 can define distinct T helper (Th) phenotypes (27). Following this concept, Th1 cells are usually defined as a population with CXCR3^+^CCR6^−^, whereas CXCR3^−^CCR6^−^ are hallmarks of Th2 cells. Recently, a new CXCR3^+^CCR6^+^ CD4+ T cell subset referred to as Th1* has been described, which appears to play a critical role in mycobacterial infections in humans (28). Lastly, Th17 lymphocytes, as defined by CXCR3^−^CCR6^+^ T cell, have also been implicated in tuberculosis (TB) immune responses and pathogenesis (29).

So, we evaluated the differential expression of CXCR3 and CCR6 on T cells. We found that there were major changes in the frequencies of CD4+ T cell subsets differentially expressing CXCR3 and CCR6 following SDV-EVs treatment (Fig. 6C). We observed a substantial increase in the frequency of CXCR3^+^ and CCR6^+^ marker. We also measured ICOS-1 expression. ICOS is a costimulatory molecule in the CD28 family, whose expression is induced during the activation of CD4+ T cells. We did not observe much difference in ICOS-1 expression across the study groups (Fig. 6C). These findings suggest that the frequencies of CD4+ T cell subsets differentially expressing CXCR3 and CCR6 are affected by SDV-EVs treatment.

### EV surface proteins are crucial for mediating signals to T cells

**Fig 7:**
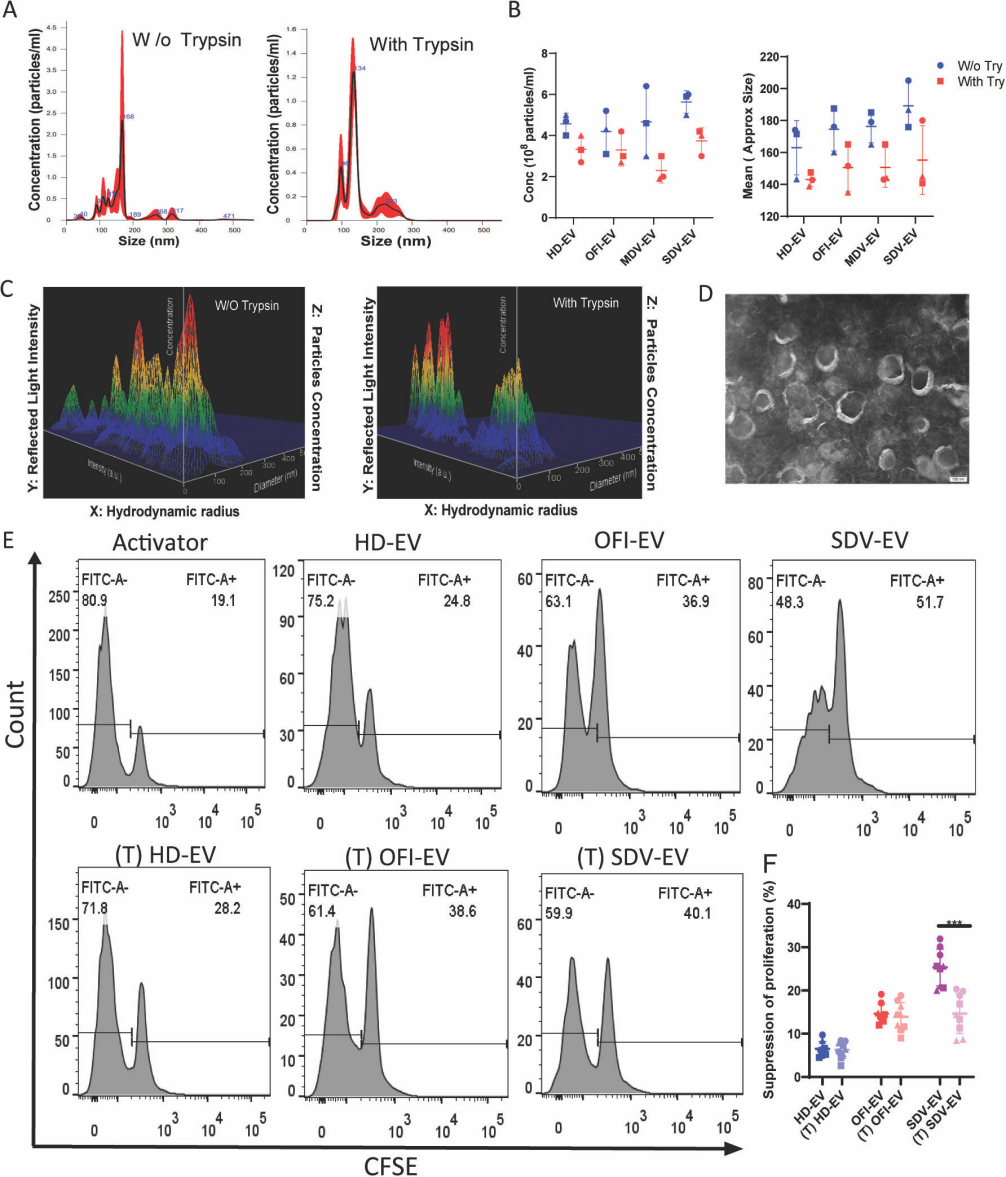
Characterization of plasma-derived EVs with or without trypsin treatment. (**A**) Size and number distribution of EVs were measured by NTA, indicating an average mean of 168 nm without trypsin-treated EVs and 134 nm of trypsin-treated EVs in all cohorts. (**B**) Combined data of size and concentration before and after the trypsinization process of EVs. Trypsinization leads to a reduction in the size and concentration of EVs. HD-EVs (*n* = 3), OFI-EVs (*n* = 3), MDV-EVs (*n* = 3), and SDV-EVs (*n* = 3). (**C**) A three-dimensional graph showing the concentration of EVs on the Z-axis. The x-axis shows the hydrodynamic radius, and the Y-axis represents reflected light intensity by the surface of EVs. (**D**) Transmission electron microscopy revealed that EVs remain intact after trypsin treatment by showing their cup-shaped morphology. (**E**) EV surface proteins are crucial for mediating the suppression of CD4+ T cells. Activated CD4+ T cells cultured with trypsinized (**T**) and control EVs derived from all four cohorts for 6 days. Representative histograms of proliferating CFSE+ CD4+ T cells incubated with EVs were shown. (**F**) Combined data of CFSE+ CD4+ proliferation on three individual healthy blood donors. A significant reduction in suppression of proliferation was observed in trypsinized (**T**) SDV-EV as compared to control SDV-EV. Suppression of proliferation was calculated by subtracting the value of the activator from the respective groups. HD-EVs (*n* = 9), OFI-EVs (*n* = 9), MDV-EVs (*n* = 9), and SDV-EVs (*n* = 9). Student unpaired two-tailed test was used to observe the *P*-value (***P = 0.0001).

To understand how EVs are capable of suppressing PBMC proliferation, we thought that it could be achieved through the interaction of surface protein, imparting negative signals to the PBMCs. When we consider the vesicle architecture, EV proteins could be categorized into three subgroups: intravesicular, plasma membrane (integral, lipid-anchored, and peripheral membrane proteins), and extravascular cargo proteins (extracellular proteins attached to EVs). To prove that extravascular proteins are important in triggering the signals, we treated the EVs with 0.25% of trypsin. Since trypsin could not penetrate through the vesicular membrane, we reason that vesicular proteins that belong to the intravesicular cargo subgroup are resistant to the trypsin treatment, while some of the vesicular cargo subgroups of the plasma membrane and extravascular cargo proteins and contaminated non-vesicular proteins are sensitive to the trypsin treatment. Treatment with trypsin led to a reduction of vesicle concentration and size (Fig. 7A and B) as observed in NTA and TEM images (Fig. 7D). Moreover, by NTA, it is possible to estimate the intensity of radiation reflected from the surface of the analyzed particles. As can be seen by comparing panel C, trypsinization leads to a significant change on the surface of EVs (Fig. 7C) (30, 31).

We did not observe much change in CD4+ proliferation status with trypsinized or un-trypsinized EVs isolated from HD or OFI plasma. However, with the trypsinized SDV-EVs, when we incubated CD4+ T cells, we found that T cell proliferation was partially restored in contrast to trypsin-untreated SDV-EVs (Fig. 7E and F), confirming that surface interaction is necessary for imparting inhibitory signals to CD4+ T cells.

### Extracellular vesicle-induced CD4+ T cell suppression occurs through PD-L1/programmed cell death 1 (PD-1) interaction

**Fig 8:**
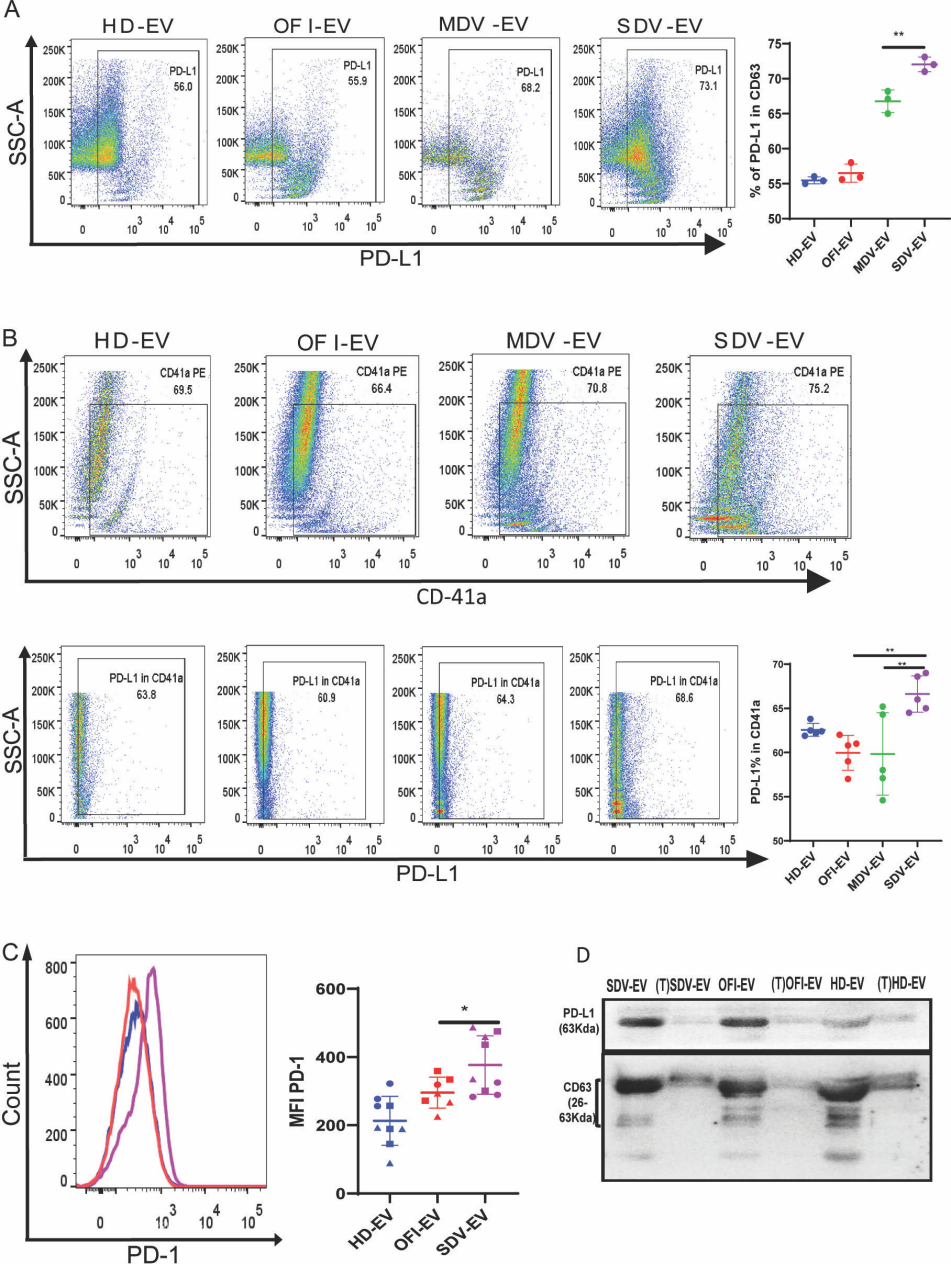
Assessment of PD-1/PD-L1 on CD4+ T cells and EVs. (**A**) Representing contour plot of the PD-L1 population in CD63 gated EVs from all four different cohorts. Quantified data are represented in the right panel (**P = 0.009). (**B**) Representing data of PD-L1 population in CD41A+ population in CD63 gated EVs from all four different cohorts. Combined data of PD-L1% in the CD41a+ population (after gating CD63) in EVs in different EV samples. HD (*n* = 5), OFI (*n* = 5), mild dengue patients (*n* = 5), and severe dengue (*n* = 5) (**P = 0.009). EVs from severe dengue (SDV-EVs) patients showed the highest percentage of PD-L1 in CD41a-gated EVs (right panel). **P = 0.007. EVs from severe dengue patients showed the highest percentage of PD-L1 in the CD41a+ population of EVs. (**C**) Representing data of PD-1 in CD4+ T cells in the presence of EVs from three cohorts. CD4+ T cells were cultured with and without EVs for 6 days in RPMI, and CD4+ T cells showed increased MFI of PD-1 in the presence of severe dengue-derived EV. The combined data graph for PD-1 MFI in three healthy donors using HD (*n* = 9), OFI (*n* = 6), and severe dengue (*n* = 9) was shown in the right panel (*P = 0.04). (**D**) Immunoblot of PD-L1 (ligand of PD-1) on trypsin(−) and trypsin (+) EVs were detected by loading 20 µg of EVs. Trypsin-treated EVs showed a reduction in PD-L1 as compared to those without trypsin-treated EVs.

The PD-1 receptor (CD279) and its ligands (PD-L1) interaction is a potent T cell inhibitor with a critical role in peripheral tolerance, but it can also compromise anti-viral T cell responses. It was recently reported that human platelets express PD-L1 (32–34). Platelets get dysregulated during severe dengue, and EVs from severe dengue patients’ plasma mostly originated from platelets (Fig. 2A). Therefore, we examined the expression of PD-L1 on the EVs. As shown in Fig. 8A, between mild and severe dengue groups, PD-L1 expression significantly enhanced in SDV-EVs (Fig. 8A), suggesting that MDV-EV- and SDV-EV-treated CD4+ T cells are exhausted T cells and thus probably less responsive to proliferation. Furthermore, PD-L1 was significantly enhanced in CD41a+ EVs isolated from severe dengue plasma compared to mild, HD, or OFI groups (Fig. 8B). Moreover, when we incubated the EVs with CD4+ T cells, we observed that compared to HD and OFI-EVs, SDV-EVs induced a significant increase in PD-1 expression on CD4+ T cells (Fig. 8C). Naïve T cells expressed low levels of PD-1 after 24 hours in culture. EVs from OFI and SDV increased the frequency of PD-1 expression on late-activating CD4+ T cells as compared to HD-EVs (Fig. 8C).

Furthermore, an equivalent number of intact particles and particles treated with trypsin was used for the analysis of exosome marker CD63 and PD-L1, by Western blotting (Fig. 8D). As expected, incubation with trypsin led to the destruction of PD-L1 but partially affected the content of transmembrane tetraspanin CD63.

**Fig 9:**
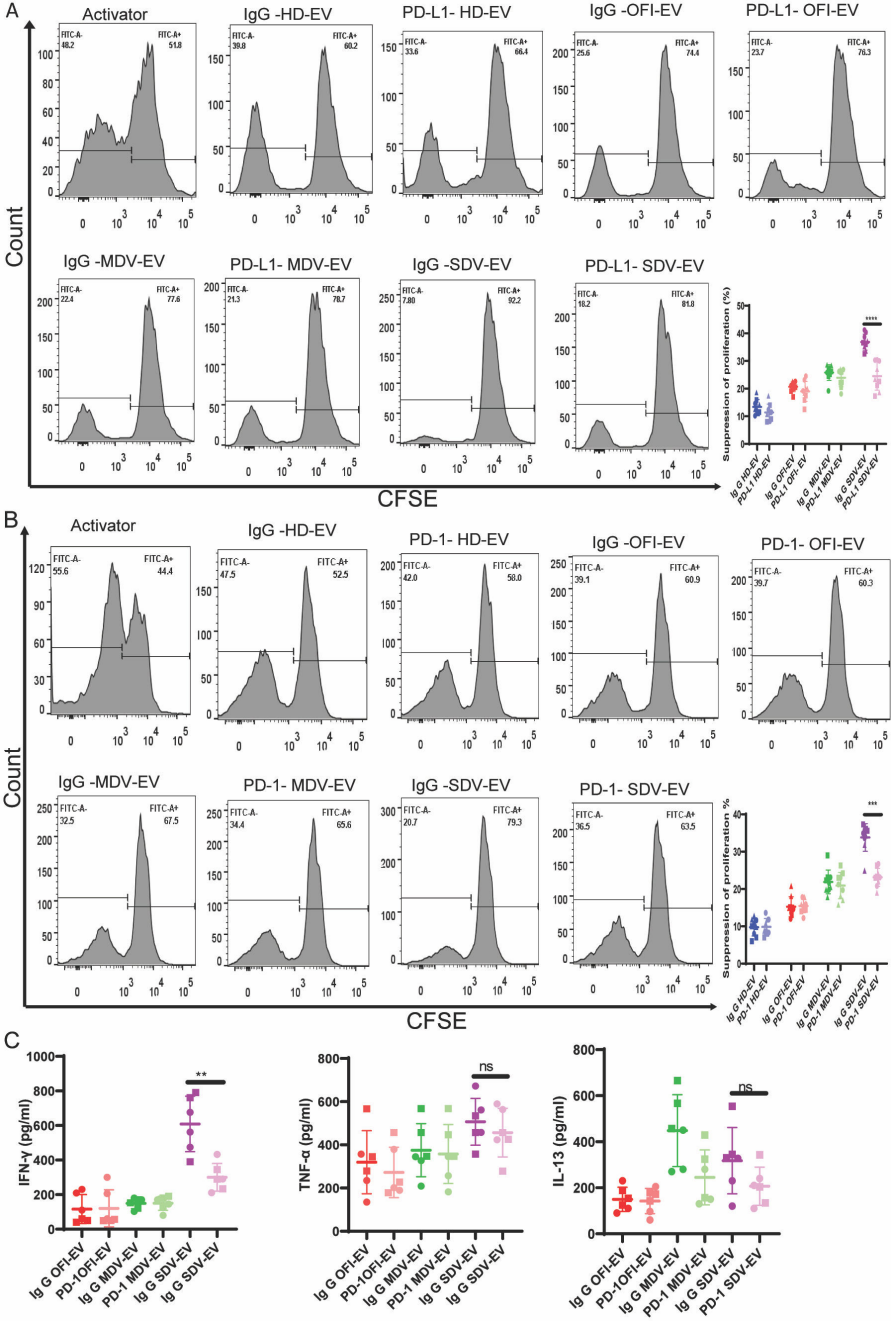
PD-L1 is crucial for mediating suppression in T cell proliferation. (**A**) Representative data of proliferating CFSE+ CD4+ T cells incubated with IgG isotype control EVs or PD-L1-blocked EVs. Activated CD4+ T cells cultured with IgG isotype control-treated EVs behaved like without trypsin-treated EVs on CD4+ T cells maintaining their suppressive capability, whereas PD-L1-blocked EVs showed a partial reduction in the suppression of proliferation of CD4+ T cells (left panel). The combined data graph of these IgG and PD-L1-blocked EVs on three individual donors was shown in the right panel. HD (*n* = 9), OFI (*n* = 9), mild dengue patients (*n* = 9), and severe dengue (*n* = 9) (****P < 0.0001). (**B**) Representative data of proliferating PD-1-blocked and IgG-treated CFSE+ CD4+ T cells incubated with EVs. IgG-treated CD4+ T cells behaved similarly as without trypsinized EVs; however, PD-1-blocked CD4+ T cells in the presence of SDV-EV showed a reduction in suppression. The combined data graph of these IgG and PD-1-blocked CD4+ T cells with three individual donors is shown in the right panel. HD (*n* = 9), OFI (*n* = 9), mild dengue patients (*n* = 9), and severe dengue (*n* = 9). Student *t*-test is used for analysis between IgG and PD-1-blocked SDV-EV groups (***P < 0.0001). (**C**) Cytokine assessment of IgG and PD-1-blocked CD4+ T cells in the presence of OFI-EV, MD-EV, and SDV-EV showed a significant reduction in IFNγ secretion in PD-1-blocked CD4+ T cells compared with IgG-treated CD4+ T cells incubated with SDV-EV. The concentration of TNF- and IL-13 is plotted. Student unpaired *t*-test is used to calculate the significance among IgG- and PD-1-treated CD4+ cells in the presence of SDV-EV (**P = 0.0018).

Next, to verify whether PD-L1 is important for inducing suppression in CD4+ T cells, we simultaneously preincubated the EVs with anti-PD-L1 antibodies to block PD-L1 expression. Along with activator (CD3:CD28), PD-L1 blocked EVs were used to treat CD4+ T cells. In another set of experiments, purified CD4+ T cells were preincubated with PD-1 to block PD-1 expression on CD4+ T cells. PD-1-blocked and CFSE-labeled CD4+ T cells were seeded, and EVs from all groups were added with activator. After 6 days post incubation, the extent of CD4+ proliferation was measured by FACS in both experiments. As shown in Fig. 9A, PD-L1 antibody treatment in EVs isolated from HD, OFI, or mild dengue plasma did not affect proliferation, and a similar extent of proliferation was noted with IgG-treated EVs. However, the percentage of CD4+ T cell suppression was significantly reduced in PD-L1 antibody-treated EVs isolated from severe dengue plasma, suggesting that EV-induced CD4+ suppression was mediated through PD-L1-PD1 interaction (Fig. 9A, right panel). In another set of experiments, PD-1-blocked CD4+ T cells seeded with HD-EVs, OFI-EVs, and MDV-EVs did not show much effect on the proliferation of CD4+ T cells, whereas PD-1-blocked CD4+ T cells when treated with SDV-EVs showed a significant reduction in the suppression of CD4+ T cells (Fig. 9B). These data were further corroborated with the cytokine level in which IFNγ levels decreased significantly in SDV-EV-treated PD-1-blocked cells. Interestingly, no significant change was observed in IL-13 and TNF-α levels (Fig. 9C). Thus, our data suggested that blocking of PD-1 on CD4+ T cells reduced SDV-EV-induced IFNγ production. Multiple studies suggest that the role of IFNγ is crucial in regulating the PD-L1 expression via the IFNγ/PD-L1 axis (35–37).

## DISCUSSION

The circulating EVs play an important role in mediating immune response and disease outcome. The goal of this study was to shed light on the role of plasma EVs released by dengue-infected cells in the regulation of immune responses in dengue patients. Our study highlights several important observations (1). SDV-EVs are not different in size from other EVs but have significantly increased numbers as compared to OFI and mild dengue (2). Platelets are the major contributors of EVs in the plasma of severe dengue patients (3). SDV-EVs carry an increased amount of many pro- and anti-inflammatory cytokines, inducing apoptosis in CD4+ cells (4). SDV-EVs induced CD4+ T cell proliferation suppression via PDL-1/PD-L1 interaction.

To define the nature and origin of the microvesicles isolated from blood plasma and to distinguish whether they come from different cellular origins, we investigated cell-specific molecules. CD41a (GPIIb) was a good candidate because it is specifically expressed by the megakaryocytic lineage. Also, the CD3 marker was used to confirm whether EVs originated from human CD8+ or CD4+ T lymphocytes. Since all EVs contain proteins derived from the parent cell and can partly maintain parent cell functionality, we characterized EV origin using antibodies against major immune cell surface receptors.

What we observed is that the source of the majority of EVs from severe dengue patients is platelet, whereas, in the mild or OFI cases, EVs mostly originated from T cells and a minority from platelets. Platelet-derived extracellular vesicles (PEVs) are produced by activated platelets in response to various activating stimuli including dengue and other viral infections (38–40). PEVs possess CD41a and CD62P and play roles in many different conditions, including in inflammation, viral infection, and immunologic responses (41).

It is corroborated by the information that more platelets get activated during severe dengue, leading to thrombocytopenia (42). Platelet degranulation is an important marker for platelet activation. The protein composition of plasma vesicles is also in agreement with their exosomal origin. In our mass spectrometry study, we observed that most of the proteins in SDV and OFI- EVs were found to be platelet originated. Further analysis suggested that unique proteins were found to be involved in platelet degranulation and negative regulation of the immune process. Increased degranulation can lead to platelet destruction. On the other hand, it increases the release of EVs. Thus, despite of decrease in platelet count, the platelet-associated EVs number increased in severe dengue.

Furthermore, in terms of cytokine content, our findings strongly support dengue plasma EVs as a risk factor able to intensify inflammation through stimulation of the proinflammatory response of peripheral blood immune cells. We found that SDV-EVs carry both pro- and anti-inflammatory cytokines, especially increased levels of IFNγ and TNF-α and IL13. Interestingly, SDV-EVs significantly induced the production of the same set of cytokines that contribute to the T cell subset proliferation in PBMCs compared with those from other EVs. Our findings are in line with these clinical observations and indicate that dengue-associated plasma EVs have the potential to contribute to excessive cytokine/chemokine responses in dengue patients. The SDV-EVs can induce apoptosis in CD4+ T cells. The exact mechanisms are currently unknown. However, increased TNF-α levels in SDV-EVs or other apoptosis inducers can trigger apoptosis in CD4+ T cells (43).

To assess whether EVs of severe dengue patients induced T cell proliferation, we cocultured EVs using allogeneic T cells derived from HDs. We observed a significant reduction in T cell proliferation. Also, these T cells had increased levels of PD-1 expression. PD-1 is a common immunosuppressive member on the surface of T cells and plays an imperative part in downregulating the immune system and advancing self-tolerance. Furthermore, PD-L1 expression was found to be significantly enhanced in SDV-EVs and its receptor PD-1 on CD4+ T cells suggesting that interaction between SDV-EVs and CD4+ T cells makes them exhausted and thus probably less responsive to proliferation.

Interestingly, the ligand of PD-1 (PD-L1) was highly detectable in EVs of severe dengue patients as compared to mild or OFI. The majority of SDV-EVs are of platelet origin. It was recently reported that human platelets express PD-L1 (32–34). It has recently been shown that inhibition of PD-L1 can suppress platelet activation (44). Platelets express PD-L1 at low levels in healthy individuals and get upregulated in cancer (45). PD-L1 gets dysregulated in COVID-19 patients; increased levels of PD-L1 were found in the serum of these patients which correlated with high lymphopenia and a high amount of C-reactive protein (CRP) (46). In HIV patients’ plasma, the level of PD-L1 in plasma correlated with plasma cytokines like TNF-α, IL-10, and IFNγ (47). Platelet activation results in the release of EV-containing PD-L1. We have previously shown that the high DV genome copies in platelets correlated directly with elevated platelet activation (48). Moreover, platelet activation status is an important determinant of thrombocytopenia in dengue infections (42). Increased levels of EVs containing PD-L1 further correlated with increased platelet activation. Increased PD-1/PD-L1 axis results in immune paralysis in the host in several viral infections like HIV, hepatitis C virus (HCV), COVID-19, and sepsis disease.

In conclusion, we show significant suppression of CD4+ T cell proliferation by SDV-EVs. Our results identify an increased level of PD-L1 in the SDV-EVs, which may contribute to T cell suppression and dengue disease progression.

## MATERIALS AND METHODS

### Characterization of EVs

#### Isolation of EVs from the ultracentrifugation process

Plasmas from mild, severe, and other febrile illnesses and healthy donors were collected individually. For EV isolation, five samples (200 µL each) were pooled to make 1-mL plasma and diluted with filtered phosphate buffer saline (PBS) to make a total volume of 11 mL. EV isolation was performed by a multistep ultracentrifugation process. Briefly, cell debris was removed by centrifugation at 2,000 rpm for 30 min and ultra-centrifuged at 20,000 × *g* for 2 hours to remove cellular fractions. The supernatant was then ultra-centrifuged at 2,00,000 × *g* by using P40ST swinging rotors (Beckman Coulter, Brea, CA, USA) for 2 hours at 4°C to obtain extracellular vesicles free from any cellular debris.

### Nanoparticle tracking analysis

The EVs were diluted (1:1,000) in filtered 1× PBS and injected into a laser chamber for NTA by NanoSight LM20 (Malvern Instruments Company, NanoSight, Malvern, UK). The following settings were used for data acquisition: camera level, 11; acquisition time, 60 seconds; and detection threshold, 3. The Brownian motion of each particle was tracked between frames thrice, and the size was calculated using the Strokes–Einstein equation.

### Transmission electron microscopy

Five microliters of EVs were placed on Formvar/carbon-coated pre-glow discharged copper grids (#CF300-CU/50) and allowed to adsorb for 2 min in a dry environment. The grid was then washed in RNAase-free water thrice for 20, 40, and 60 seconds; stained with 1.5% phospho-tungstic acid for 50 seconds; and placed in a clean environment for drying. The grid was observed at the 80 k threshold under the transmission electron microscope (JEOL-JEM 1400 flash, JEOL, USA).

### Western blotting

The protein content and concentration of the isolated extracellular vesicles were determined using the micro-BCA protein assay kit (# 23235, Thermo Pierce, Rockford, IL, USA) following the manufacturer’s instructions. Briefly, EVs were lysed in Lane Marker Reducing Sample Buffer containing DTT, SDS, and B-mercapto-ethanol separated on 10% SDS-PAGE gels (Bio-Rad) always applying 20 µg protein/lane and transferred onto an Immobilon-P PVDF membrane (EMD Millipore) for Western blotting. Membranes were blocked with 5% bovine serum albumin (BSA) followed by overnight incubation at 4°C with different antibodies of tetraspanins present in EVs. The following antibodies were used: CD81 SC23962, CD9 SC13118 (Santa Cruz, CA, USA), Alix mAB # 2171, and CD63 AB 216130 (Abcam, Cambridge, UK). Horseradish peroxidase (HRP)-conjugated secondary antibody was incubated for 1 hour at room temperature (RT), and after giving three washes of PBST, blots were developed by using an ECL detection kit (# 32132 Thermo, Waltham, USA)

### Cytokine profiling of plasma EVs

The protein levels of various pro-inflammatory cytokines, IFNγ, TNF-α, IL-6, IL-2, and IL-17a, and anti-inflammatory cytokines, IL-13, IL-5, IL-22, IL-10, and IL-4, were measured in plasma and EVs using legend plex (#741027, BioLegend, San Diego, USA). EVs (20 µg) and plasma samples were diluted with legend Plex sample diluent/assay buffer, and samples were prepared for the cytokine analysis according to the manufacturer’s instructions. The concentration (pg/mL) of 12 different cytokines was measured using flow cytometry, and data were analyzed by oognit software (BioLegend San Diego, USA).

### Source of EVs

To detect the source of EVs derived from these four groups, flow cytometry was done by using 4-µm aldehyde/latex beads (#A37304, Thermo, Waltham, MA, USA). They were incubated with these beads in 5 µL for 20 µg of EV concentration overnight and centrifuged, and excess beads were removed. Ten percent BSA was added to block unspecific sites for 30 min as described in reference 49 and then centrifuged at 15,000 rpm for 30 min to remove BSA and diluted in filtered PBS. Then, the antibody of CD63-BV421 (#740080, BD Biosciences, San Diego, USA), CD41A-PE-CY7 (#2384755, Invitrogen, Waltham, USA), and CD3-APC (#300318, BioLegend, San Diego, USA) were added and acquired using FACS Verse on the low threshold. Data were analyzed by FlowJo v10 (FlowJo LLC).

### Mass spectrometry

Equal concentrations of EVs samples were trypsinized followed by sample clean-up with Waters Oasis SPE cartridge, and vacuum-dried peptides were resuspended in 10-µL 2% ACN in 0.1% FA and subjected to MS/MS using TripleTOF 5600+ (ABsciex) mass spectrometer instrument. The mass analyzer was attached through the trap and analytical column with specification ChromeXP, 3C18-CL-120, 3 µm, 120 Å, and 0.3 × 150 mm, respectively. The spray nozzle was connected with an electro-ionization source to inject the peptide sample into a mass spectrometer. The used flow rate was 5 µL/min for the analytical chromatography to separate the peptides in the continuous gradient of elution with the 2%–90% acetonitrile for 87 min total run time. The system uses a solvent composition with a mixture of two reservoirs. Reservoir A with 98% water and 2% ACN in 0.1% FA and B with 98% ACN and 2% water in 0.1% FA were used.

For label-free quantification (SWATH analysis), the data-independent acquisition (DIA) mode was applied. In the SWATH acquisition method, the Q1 transmission window was set to 12 Da from the mass range 350–1,250 Da. A total of 75 windows were acquired independently with an accumulation time of 60 ms; the total cycle time was kept constant at 4.75 s. The ion source was operated with the following parameters: ion spray voltage floating (ISVF) = 5,500; gas source 1 (GS1) = 25; GS2 = 22; curtain gas (CUR) = 30.

### MS data processing and analysis

Three technical replicates for each of the sets were analyzed. The raw data were loaded in Spectronaut X software version 12.0.2 (Biognosys), and data analysis was performed using the directDIA method, against the UniProt-Human database.

### CFSE-based PBMC proliferation assay

Blood was collected in an EDTA vial with prior consent and diluted with an equal volume of DPBS (catalog no. 2381367, Thermo Waltham, MA, USA). PBMC of healthy donors isolated by Lymphoprep (STEMCELL, #07851, Vancouver, Canada) layering on sepmate tube (catalog no. #15415, STEMCELL, Vancouver, Canada) and centrifuged at 800 × *g* for 20 min with break off. The lymphocyte layer was collected and washed twice with DPBS at 300 × *g* for 10 min. Isolated lymphocytes were labeled with 0.5 µM CFSE (Cell Trace, #C34554, Thermo Scientific, Waltham, MA, USA) in DPBS for 10 min at 37°C. The excess staining was removed with an equal volume of 10% FBS (exosome depleted) RPMI and centrifuged twice at 800 rpm for 10 min. Obtained cells were counted and seeded (0.2 * 10^6^) in 96 U-bottom well plate, activated with CD3:CD28 (Immunocult STEMCELL #10991, Vancouver, Canada), and added with EVs at 20-µg protein concentration and incubated for 6 days. The proliferation of lymphocytes was measured by flow cytometry, and the data were analyzed by FlowJo v10 (FlowJo LLC).

### Detection of CD4+ and CD8+ T cells in PBMC

Blood was collected in an EDTA vial and diluted with an equal volume of DPBS. PBMC of healthy donors isolated by Lymphoprep layering and centrifuged at 800 × *g* for 20 min with break off. The lymphocyte layer was collected and washed twice with DPBS at 300 × *g* for 10 min. Obtained cells were counted and seeded (0.2 × 10^6^) in 96 U-bottom well plate, activated with CD3:CD28 (25 µL/mL) and with EVs at 20-µg protein concentration, and incubated for 6 days. On the sixth day, cells were harvested by centrifuging them at 1,200 rpm for 10 min; brilliant stain buffer was added containing CD3 APC H7 (#300318, BioLegend, San Diego, USA), CD4 BV480 (#317444, BioLegend, San Diego, USA), and CD8 PE (#2436995, Invitrogen, Waltham, USA). Levels of CD4+ and CD8+ T cells were detected by flow cytometry. CD3+-gated cells were used for further analysis by FlowJo v10 (FlowJo LLC).

### Apoptosis of CD4+ T cells

CD4+ T cells isolated from PBMC of healthy donors’ blood by stem cell CD4+ isolation kit (catalog no. 17952, STEMCELL, Vancouver, Canada) counted and seeded (0.2 × 10^6^) in 96 U-bottom well plate, activated with CD3:CD28 (25 µL/mL) and with EVs at 20-µg protein concentration, and incubated for 24 hours and 6 days. After 24 hours and on the sixth day, cells were harvested by centrifuging them at 12,000 rpm for 10 min at 4°C, and resuspended the pellet was in annexin binding buffer, and annexin-FITC, 7-AAD was added as per manufacturers guidelines (catalog no. 640922, BioLegend). Flow cytometry recorded 50k cell events.

### CFSE labeling of EVs

EVs from different groups were taken in 200-µg protein concentration and labeled with 0.2 µM CFSE (#C34554, Cell Trace, Thermo Scientific) for 10 min and then centrifuged at 200,000 × *g* for 1 hour to remove excess dye. These labeled extracellular vesicles were added with CD4+ T cells at different time points in 96 U-bottom plates in EV-depleted RPMI media to observe their uptake (34).

### CFSE-based CD4+ proliferation assay

CD4+ T cells isolated from PBMC of healthy donors’ blood by stem cell CD4+ isolation kit (#17952 STEMCELL, Vancouver, Canada) based on negative selection were labeled with 0.5 µM CFSE (#C34554, Cell Trace, Thermo Scientific) in DPBS for 10 min at 37°C. The excess staining was removed with an equal volume of 10% FBS (exo-depleted) RPMI. CFSE-labeled T cells (10^5^ cells/well) were activated with CD3/28 beads (25 µL/mL, T cell activation/expansion kit, STEMCELL, Vancouver, Canada) and co-incubated with EVs in 20-µg concentration for 6 days at 37°C. On the sixth day, cells were harvested by centrifuging them at 1,200 rpm for 10 min; proliferation of T cells was measured on day 6 by flow cytometry, and the data were analyzed by FlowJo v10 (FlowJo LLC).

### Subsets of CD4+ T cells

Isolated T cells were activated with CD3:CD28 and incubated with EVs at a concentration of 20 µg from three groups for 6 days at 37°C. On the sixth day, cells were harvested by centrifuging them at 1,200 rpm for 10 min, and their media supernatant was stored for further detection of cytokines in them by legend-plex CBA kit (#741027, BioLegend, San Diego, CA, USA); brilliant stain buffer was added containing CXCR3 PE (#562152, BD Biosciences, San Diego, USA), CCR6 BB515 (#564479, BD Biosciences) ICOS-1 AF647, and PD-1 BV421 (#AB205921, BD Biosciences, San Diego, USA). A total of 50,000 events were acquired in flow cytometry.

### Cytokine profiling of CD4+ T cell supernatant

The protein levels of various pro-inflammatory (IFNγ, TNF-α, IL-6, IL-2, and IL-17a) and anti-inflammatory (IL-13, IL-5, IL-22, IL-4, and IL-10) cytokines in CD4+ T cells supernatant were measured using legendplex (#741027, BioLegend, San Diego, USA). Samples at 20-µg protein concentration diluted with assay buffer were used as samples for the detection. The concentration (pg/mL) of 12 different cytokines was measured using flow cytometry and data were analyzed by oognit software (BioLegend).

### Trypsinization of EVs

A 200-µg protein equivalent of isolated EVs was incubated with 50 µg/mL of trypsin for 6 hours at RT on continuous shaking to remove surface proteins. Excess trypsin was removed by ultracentrifugation at 200,000 × *g* by using P40 ST (Beckman Coulter). Control EVs underwent the same procedure as trypsin-treated EVs in the absence of trypsin as described earlier (30).

### Blocking of PD-L1 on EV membrane and PD-1 on CD4+ T cells

To block PD-L1 on the EV surface, purified EV samples (200 µg protein equivalent) were incubated with PD-L1 blocking antibodies (10 µg/mL) or IgG isotype antibodies (10 µg/mL) in 200 µL PBS and then washed with 10 mL PBS and pelleted by ultracentrifugation to remove the non-bound free antibodies. Then, these EVs were used on CD4+ T cells to check the effect of PD-L1 by CFSE Proliferation assay as described earlier (36). Purified CD4+ T cells were incubated with PD-1 blocking antibody (10 μg/mL) ( #149989-80, Thermo Pierce, Rockford, IL, USA) or IgG isotype antibody (10 µg/mL) in 200 µL of PBS for 2 hours at RT. For removing excess PD-1, washing was done twice, and the pellet was resuspended in DPBS; labeling of CD4+ was done by using CFSE, and excess CFSE was removed by washing. The labeled pellet was resuspended in 10% exo-depleted FBS containing RPMI, and 0.2 × 10^6^ cells were seeded on a 96 U-bottom plate. Activator and EVs were added to the respective wells. Cells were harvested after 6 days for analysis of proliferation through FACS, and supernatant of PD-1 blocked CD4+ T cells was collected and assessed for cytokine profiling. The supernatant (20-µg protein equivalent) was used for the CBA through FACS.

### Statistical analysis

Each experiment is performed on three individual healthy donors with three sets of different EVs to obtain the pattern and to have reduced variability. Every dot represents the data of one donor by the one set of EV samples. Analysis of variance with multiple comparisons and unpaired student *t*-tests are mainly used for the comparative perspective. Statistical analysis was performed using GraphPad Prism, and FACS analysis was done by FlowJo X software. A *P* value of <0.05 was considered statistically significant.

## Data Availability

The mass spectrometry proteomics data have been deposited to the ProteomeXchange Consortium via the PRIDE [http://www.ebi.ac.uk/pride] partner repository with the dataset identifier PXD045894.
